# Supplementary material for: Probing solution structure of the pentameric ligand-gated ion channel GLIC by small-angle neutron scattering
Source: Proc Natl Acad Sci U S A. 2021 Sep 9;118(37):e2108006118. doi: 10.1073/pnas.2108006118 (PMC8449418; doi:10.1073/pnas.2108006118)
Supplement: Supplementary File [file pnas.2108006118.sapp.pdf]

PNAS
www.pnas.org

Supplementary Information for

Probing solution structure of the pentameric ligand-gated ion channel GLIC by small-angle neutron scattering

Marie Lycksell, Urška Rovšnik, Cathrine Bergh, Nicolai T Johansen, Anne Martel, Lionel Porcar, Lise Arleth, Rebecca J Howard, Erik Lindahl

Corresponding Author Erik Lindahl.
E-mail: erik.lindahl@dbb.su.se

This PDF file includes:

Figs. S1 to S2
Tables S1 to S6
Legends for Movies S1 to S2
SI References

Other supplementary materials for this manuscript include the following:

Movies S1 to S2

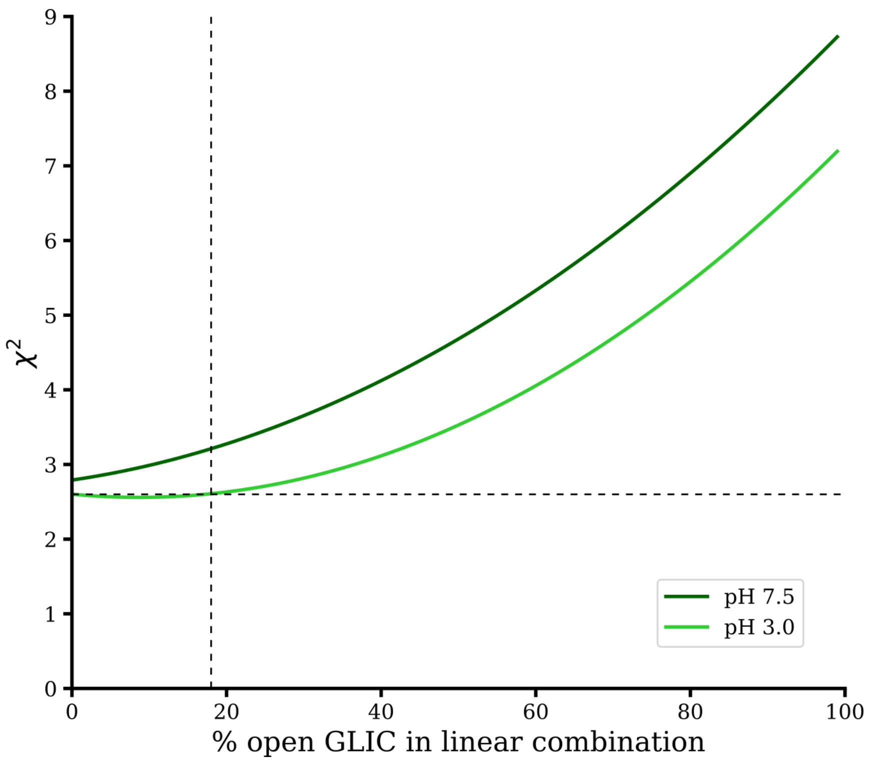

Fig. S1. Goodness of fit (χ^2) to SANS data collected under resting conditions (pH 7.5, dark green) or activating conditions (pH 3.0, light green), as a function of the contribution of the open GLIC crystal structure, in linear combination with the closed GLIC crystal structure. The linear combination when fitted to the pH 3 SANS data yields as good a fit as the closed crystal structure alone (horizontal dashed line) up to 18% contribution from the open crystal structure (vertical dashed line).

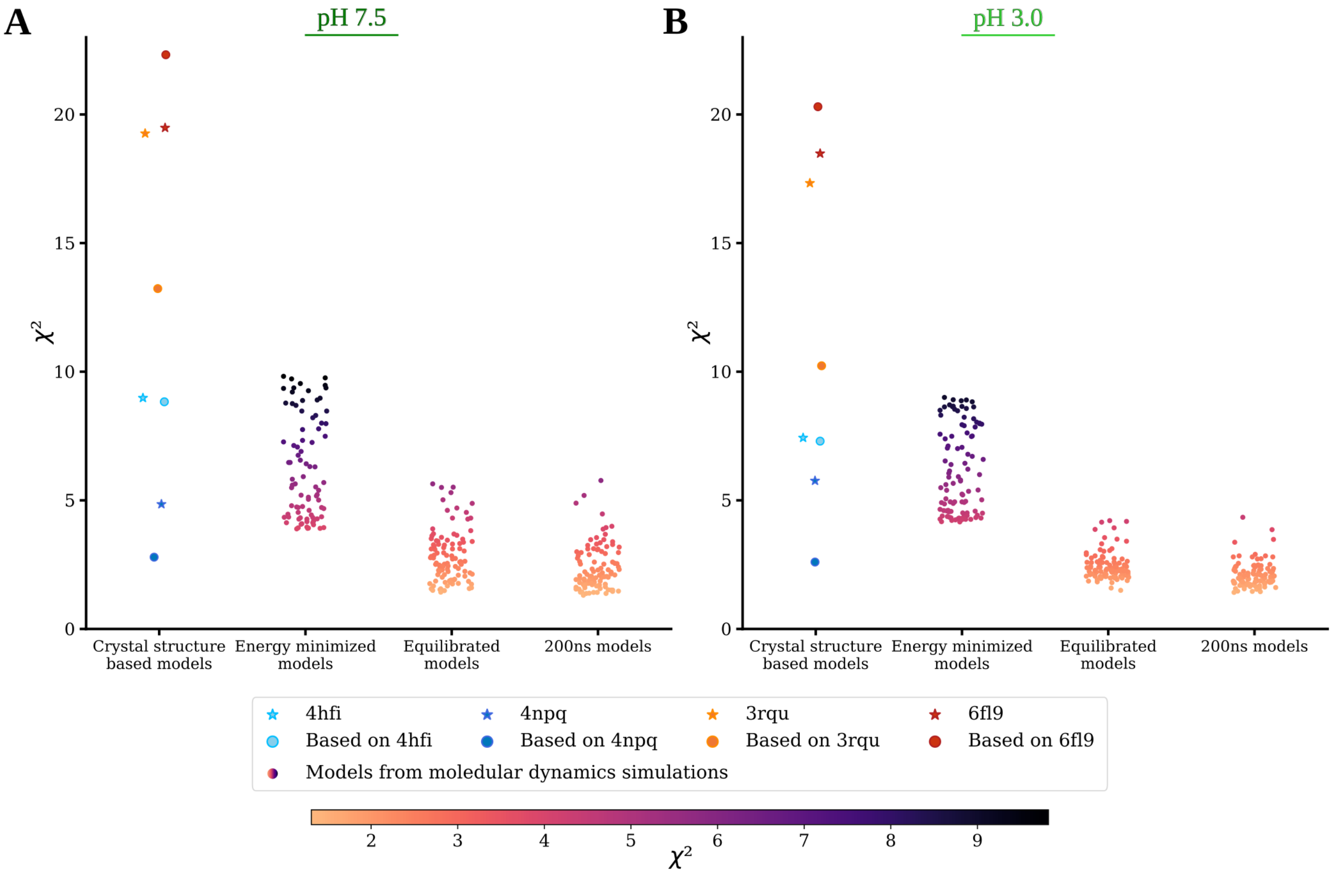

Fig. S2. Comparison of model fits (χ^2) to SANS data collected under **A)** resting (pH 7.5) or **B)** activating (pH 3.0) conditions. In the first column of each panel, crystal structures are shown as stars; models containing the full sequence of the processed GLIC construct are shown as circles, colored as for the crystal structures on which they were based. In subsequent columns, models from MD simulations are shown after energy minimization, after equilibration, and after 200 ns of unrestrained simulation, all as circles colored by χ^2 value.

Table S1. Summary of structural parameters calculated from the paused-flow SEC-SANS data. For the Guinier analysis the minimum and maximum Q-value included is also listed.

	pH 7.5	pH 3
Guinier analysis		
$I(0)$ (cm^{-1})	0.44	0.45
R_g (Å)	38.4 ± 0.2	38.3 ± 0.2
Q_{min} (Å^{-1})	0.010	0.010
Q_{max} (Å^{-1}), (QR_g max)	0.046, (1.7)	0.046, (1.7)
Coefficient of correlation R^2	0.9994	0.9995
M from $I(0)$ (kDa), (ratio to predicted)	194, (1.06)	194, (1.06)
$P(r)$ analysis		
$I(0)$ (cm^{-1})	0.441 ± .225E-03	0.445 ± .143E-03
R_g (Å)	37.98 ± 0.03	37.81 ± 0.02
d_{max} (Å)	109.50 ± 1.03	105.91 ± 0.54
q range (Å^{-1})	0.0060 - 0.2485	0.0060 - 0.2485
χ^2	1.04	0.91
PepsiSANS		
R_g (Å)	37.6	37.0

Table S2. χ^2 **for select contributions of the open GLIC crystal structure in a linear combination with the closed GLIC crystal structure. Any contribution of the open structure makes the fit to the SANS data from resting conditions (pH 7.5) worse, while for the SANS data from activating conditions (pH 3) the fit starts becoming less good that the closed crystal structure alone from 18% open structure contribution. The best fit to the activating condition is obtained for 6-11% open contribution, which all yield a** χ^2 **of 2.56.**

% open GLIC	χ^2 to pH 7.5	χ^2 to pH 3
0	2.79	2.60
5	2.88	2.57
10	2.99	2.56
15	3.12	2.58
16	3.15	2.59
17	3.18	2.60
18	3.21	2.61
19	3.24	2.62
20	3.28	2.63
25	3.45	2.71
30	3.65	2.82
40	4.12	3.12
50	4.68	3.53
75	6.47	5.06
100	8.83	7.30

Table S3. Summary of modeling using protein structures and models based on protein structures, covering the radius of gyration of the model and the χ^2 goodness of fit to the experimental data for the model.

Crystal structures	4npq	4hfi	3rqu	6fl9
PepsiSANS				
Predicted R_g (Å)	37.5	37.2	36.6	37.0
pH 7.5 χ^2	4.9	9.0	19.3	19.5
pH 3.0 χ^2	5.8	7.4	17.3	18.5
All residue models				
Based on	4npq	4hfi	3rqu	6fl9
PepsiSANS				
Predicted R_g (Å)	37.9	37.4	37.1	37.4
pH 7.5 χ^2	2.8	8.8	13.2	22.3
pH 3.0 χ^2	2.6	7.3	10.2	20.3
MD-simulation models				
eBDIMS				
Extrapolation	From 4npq to 4hfi<br>From 4hfi to 4npq			
Output conformations (nr/extrapolation)	25			
Equilibration time (ns/simulation)	76			
Simulation time (ns/simulation)	200			
In aggregate (ns)	20000			
PepsiSANS				
χ^2 range				
After energy minimization				
pH 7.5	3.9 - 9.8			
pH 3.0	4.2 - 9.0			
After equilibration				
pH 7.5	1.4 - 5.6			
pH 3.0	1.5 - 4.2			
After 200 ns simulation				
pH 7.5	1.3 - 5.8			
pH 3.0	1.4 - 4.3			

Table S4. Sample details, covering details of the protein, and experimental details like sample concentration, volume, and buffer.

Organism of origin	*Gloeobacter violaceus*
Expression system	*Echerichia coli*
UniProt ID	Q7NDN8
Extinction coefficient [A_{280} 0.1%(w/v)]	1.366
Volume from structure (Å^3)	
All-residue model based on 4npq	273150
Particle contrast from sequence and solvent constituents, $\Delta\bar{\rho}$ ($\rho_{protein} - \rho_{solvent}$; 10^{10} cm^{-2})	
pH 7.5	4.12 (2.25 - 6.37)
pH 3.0	4.14 (2.23 - 6.37)
M from chemical composition (kDa)	182.7
SEC-SANS	
Column	Superdex 200 Increase 10/300
Loading concentration (mg/ml)	
Continuous-flow SEC-SANS	4.0
Paused-flow SEC-SANS	5.6
Injection volume (µl)	
Continuous-flow SEC-SANS	300
Paused-flow SEC-SANS	240
Flow rate (ml/min)	
Continuous-flow SEC-SANS	0.3, 0.05
Paused-flow SEC-SANS	0.2, 0.01, 0
Average concentration (mg/ml) in combined data frames	
Paused-flow SEC-SANS	
pH 7.5	0.77
pH 3.0	0.74
Cuvette SANS	
Concentration (mg/ml)	0.47
Volume (µl)	360
Solvent	
pH 7.5	D_2O, 150 mM NaCl, 20 mM Tris·HCl, 0.5 mM d-DDM
pH 3.0	D_2O, 150 mM NaCl, 20 mM citrate·HCl, 0.5 mM d-DDM

Table S5. Summary the SANS collection parameters, including wavelength, pathlength, detector distances, Q-range, absolute scaling method, and normalization.

Instrument	ILL D22
Wavelength (Å)	6
Pathlength (cm)	
Cuvette SANS	0.2
Continuous-flow SEC-SANS	0.1
Paused-flow SEC-SANS	0.1
Detector distances (m)	
Cuvette SANS	2m/2.8m & 11.2m/11.2m
Continuous-flow SEC-SANS	2m/2.8m & 11.2m/11.2m
Paused-flow SEC-SANS	2.8m/2.8m & 8m/8m
Q measurement range ($Å^{-1}$)	
Cuvette SANS	0.004 - 0.457
Continuous-flow SEC-SANS	0.004 - 0.456
Paused-flow SEC-SANS	0.006 - 0.452
Absolute scaling method	Incident beam flux
Normalization	Divided by concentration
Exposure time (aggregate time)	
SEC-SANS	
Continuous-flow	44 x 30s (22min)
Paused-flow, pH 7.5	116 x 30s (58min)
Paused-flow, pH 3.0	106 x 30s (53min)
Cuvette SANS	2 x 1500s (50min)
Sample temperature (°C)	10

 Marie Lycksell, Urška Rovšnik, Cathrine Bergh, Nicolai T Johansen, Anne Martel, Lionel Porcar, Lise Arleth, Rebecca J Howard, Erik Lindahl

Table S6. Summary of software and equations employed for SANS data reduction, analysis, and interpretation.

SANS data reduction	GRASP v. 9.04 (1)
Extinction coefficient estimate	ProtParam (2)
Guinier equation	$\ln(I(Q)) = \ln(I(0)) - \frac{R_g^2}{3} Q^2$
Calculation of M_w	$M_w = \frac{N_A \cdot I(0)}{c(\Delta\rho \cdot \bar{\nu})^2}$
Calculation of ρ	$\rho = (\sum_{i=1}^{N} b_i)/V$
Calculation of $\bar{\nu}$	$\bar{\nu} = V/M_{aa}$
Protein volume estimation	^{3}V: Voss Volume Voxelator (3)
$P(r)$ analysis	BayesApp (4) *via* web server (https://somo.chem.utk.edu/bayesapp/)
$P(r)$ from structure	CaPP (5) (https://github.com/Niels-Bohr-Institute-XNS-StructBiophys/CaPP)
Atomic structure modelling	PepsiSANS v. 3.0 (6)
Missing sequence modelling	MODELLER v. 9.22 (7)
Structure extrapolation	eBDIMS (8, 9)
Molecular dynamics simulations	GROMACS v. 2018.4 and 2019.3 (10)
Theoretical R_g	PepsiSANS v. 3.0 (6)
Hydrogen-deuterium exchange	PSX (11)
Three-dimensional graphic model representation	VMD (12)
Plots	MATPLOTLIB (13)

Movie S1. The first principal component derived from alignment of 46 GLIC crystal structures as previously described (8), showing sequential projection of Cα atom positions in energy-minimized models along the component. Models are shaded dark to light blue based on similarity to closed versus open crystal structures.

Movie S2. The second principal component derived from alignment of 46 GLIC crystal structures as previously described (8), showing sequential projection of Cα atom positions in energy-minimized models along the component. Models are shaded dark to light blue based on similarity to closed versus open crystal structures.

References

1. C Dewhurst, *GRASP v. 9.04.* (Institute Laue-Langevin), (2020-08-24).
2. E Gasteiger, et al., Protein identification and analysis tools on the expasy server in *The proteomics protocols handbook.* (Springer), pp. 571–607 (2005).
3. NR Voss, M Gerstein, 3v: cavity, channel and cleft volume calculator and extractor. *Nucleic acids research* **38**, W555–W562 (2010).
4. S Hansen, Bayesapp: a web site for indirect transformation of small-angle scattering data. *J. Appl. Crystallogr.* **45**, 566–567 (2012).
5. AH Larsen, Capp: Calculating pair distance distribution functions for proteins (2020).
6. S Grudinin, *Pepsi-SANS v. 3.0.* (Nano-D team, Inria/CNRS Grenoble), (2020).
7. B Webb, A Sali, Comparative protein structure modeling using modeller. *Curr. Protoc. Bioinforma.* **54**, 5.6.1–5.6.37 (2016).
8. L Orellana, O Yoluk, O Carrillo, M Orozco, E Lindahl, Prediction and validation of protein intermediate states from structurally rich ensembles and coarse-grained simulations. *Nat. communications* **7**, 1–14 (2016).
9. L Orellana, J Gustavsson, C Bergh, O Yoluk, E Lindahl, ebdims server: protein transition pathways with ensemble analysis in 2d-motion spaces. *Bioinformatics* **35**, 3505–3507 (2019).
10. MJ Abraham, et al., Gromacs: High performance molecular simulations through multi-level parallelism from laptops to supercomputers. *SoftwareX* **1**, 19–25 (2015).
11. MC Pedersen, et al., Psx, protein–solvent exchange: software for calculation of deuterium-exchange effects in small-angle neutron scattering measurements from protein coordinates. *J. Appl. Crystallogr.* **52**, 1427–1436 (2019).
12. W Humphrey, A Dalke, K Schulten, VMD – Visual Molecular Dynamics. *J. Mol. Graph.* **14**, 33–38 (1996).
13. JD Hunter, Matplotlib: A 2d graphics environment. *Comput. Sci. & Eng.* **9**, 90–95 (2007).
